# Predicting Recurrence Risk of Glioblastoma Based on Preoperative-Postoperative Longitudinal MRI: A Multicenter Study

**DOI:** 10.3390/bioengineering13060668

**Published:** 2026-06-09

**Authors:** Chengwei Chen, Fan Guo, Dong Huang, Yao Zheng, Yuefei Feng, Tianci Liu, Yuxuan Yao, Jie Wei, Minwen Zheng, Yang Liu

**Affiliations:** 1 School of Biomedical Engineering, Air Force Medical University, No. 169 Changle West Road, Xi’an 710032, China; 2 Department of Radiology, Xijing Hospital of Air Force Medical University, No. 169 Changle West Road, Xi’an 710032, China; 3 Shaanxi Provincial Key Laboratory of Bioelectromagnetic Detection and Intelligent Perception, No. 169 Changle West Road, Xi’an 710032, China; 4 Innovation Research Institute, Xijing Hospital of Air Force Medical University, No. 169 Changle West Road, Xi’an 710032, China

**Keywords:** brain tumor, glioblastoma, magnetic resonance imaging, preoperative-postoperative longitudinal analysis, recurrence risk prediction

## Abstract

Glioblastoma has a high recurrence rate, yet conventional single-time-point imaging fails to capture the dynamic tumor evolution before and after surgery. This study aims to develop a deep learning model based on preoperative and postoperative longitudinal MRI to predict postoperative recurrence risk by capturing imaging dynamics. We propose MambaDiff-Net, which employs a dual-stream encoder to extract multi-scale features from preoperative and postoperative T2WI. It also includes a feature discrepancy computation module to model longitudinal imaging changes, outputting individualized recurrence risk probabilities. We included 139 patients with glioblastoma (59 training, 40 internal validation, 40 external test), with recurrence within 6 months post-surgery as the prediction target. Performance was evaluated using AUC, accuracy, and F1. MambaDiff-Net achieved AUCs of 0.887 and 0.762 in internal and external validation, respectively, significantly outperforming single-time-point models. Kaplan-Meier analysis demonstrated effective risk stratification, and decision curve analysis confirmed superior clinical net benefit. Grad-CAM visualization showed the model’s focus shifting from preoperative tumor parenchyma to postoperative resection cavity margins, consistent with clinical knowledge. A deep learning model based on preoperative-postoperative longitudinal MRI can accurately predict postoperative recurrence risk in glioblastoma. By modeling dynamic imaging changes before and after surgery, it supports individualized treatment decisions.

## 1. Introduction

Glioblastoma represents the most malignant subtype of glioma, with a postoperative recurrence rate exceeding 90% within 1–2 years [1,2,3]. Recurrent tumors are generally more invasive and proliferate more rapidly, leading to a marked shortening of patients’ subsequent survival [4]. This high recurrence rate is closely linked to the distinctive recurrence pattern of glioma [5]. Clinical observations have shown that glioblastoma recurrence is not an abrupt event but a continuous dynamic process that begins preoperatively and evolves postoperatively [6,7]. Tumor tissues harbor subpopulations of cells with high invasive potential prior to surgery. Although surgical resection removes most of the macroscopically visible tumor mass, some cellular subpopulations persist microscopically within the surgical cavity, gradually proliferating and invading, ultimately evolving into radiographically detectable recurrent lesions [8,9]. Consequently, accurately capturing recurrence risk signals along the preoperative-to-postoperative evolutionary trajectory remains a critical unresolved challenge.

Magnetic resonance imaging (MRI) is used throughout the entire process of glioma diagnosis and treatment and serves as the core imaging modality for postoperative follow-up and recurrence monitoring, as recommended by clinical guidelines [10,11]. With the advancement of radiomics and deep learning technologies, quantitative analysis has provided powerful tools for mining recurrence-related features in MRI images that are imperceptible to the naked eye [12,13]. Numerous studies have confirmed that MRI-derived imaging features can effectively reflect tumor biological behavior: texture features and heterogeneity parameters extracted from preoperative images are closely associated with patient prognosis [14,15,16]; whereas analysis of enhancing regions and edema extent on postoperative images can aid in distinguishing true recurrence from treatment-related effects [17,18]. However, existing studies share a critical limitation: most methods are confined to single-time-point static imaging analysis, which fails to reflect the dynamic changes in tumor imaging features before and after surgery, nor can it characterize the potential trend of recurrence through temporal feature changes, essentially neglecting the continuous nature of glioblastoma recurrence [19,20]. In fact, existing studies have shown that the ability to describe tumor phenotypic evolution by integrating longitudinal imaging data is significantly superior to single-time-point analysis, suggesting that incorporating temporal imaging biomarkers into standardized monitoring protocols holds substantial value and provides a feasible direction for overcoming current research limitations [21,22,23].

In clinical practice, the six-month postoperative time point serves as an important prognostic landmark. On one hand, tumor progression occurring early after surgery often indicates a worse clinical outcome, as these patients exhibit significantly shorter survival compared to those with late recurrence, and thus early progression is recognized as an independent negative prognostic factor [24,25]. On the other hand, six-month progression-free survival (PFS6) is currently widely adopted as a surrogate endpoint for treatment efficacy evaluation in phase II clinical trials of glioblastoma [26,27,28]. According to the RANO 2.0 criteria, events occurring between three and six months have largely excluded the confounding effect of pseudoprogression, thereby enabling a more reliable reflection of true tumor recurrence status [29].

Therefore, this study proposes a deep learning model based on preoperative and postoperative longitudinal MRI to accurately predict the risk of postoperative recurrence in glioblastoma, particularly to identify high-risk patients within six months after surgery. The model employs a dual-stream encoder to extract multi-scale features from dual-time-point images and uses a feature discrepancy computation module to model the dynamic changes in longitudinal imaging, outputting an individualized recurrence risk probability. This approach aims to achieve a transition from static analysis to dynamic risk assessment, supporting individualized clinical decision-making and follow-up management.

## 2. Materials and Methods

### 2.1. Research Cohort

This was a multicenter retrospective study. We used a private dataset termed XJYY-GBM (collected from Xijing Hospital, the First Affiliated Hospital of the Air Force Medical University), along with two public datasets (LUMIERE and RHUH-GBM). The study protocol was approved by the Medical Ethics Committee of Xijing Hospital (the First Affiliated Hospital of the Air Force Medical University), which granted a waiver of informed consent (Approval No. KY20243576-1). The use of public datasets (LUMIERE and RHUH-GBM) complied with their respective data usage agreements and ethical guidelines. All patient information was anonymized prior to analysis. This study strictly adhered to the Declaration of Helsinki and relevant human subject protection regulations.

This study included three longitudinal MRI datasets of glioblastoma, with acquisition parameters covering different magnetic field strengths and scanner vendors. The public LUMIERE dataset was primarily acquired on 1.9–2.0 T scanners (Siemens Healthineers, Erlangen, Germany), including T1, T1CE, T2, and FLAIR sequences, with slice thicknesses from 1.25 to 3.8 mm and in-plane resolutions from 0.6 to 0.9 mm. The public RHUH-GBM dataset was scanned at 1.5 T, comprising T1WI, T1CE, T2WI, FLAIR, and DWI sequences, with slice thicknesses ranging from 1 to 5 mm and matrix sizes from 128 × 160 to 512 × 512. The internal XJYY-GBM dataset was a multicenter retrospective cohort acquired mainly on 1.5 T and 3.0 T Siemens scanners, with a field of view (FOV) of 220–256 mm and matrix dimensions ranging from 256 × 256 to 320 × 320. Collectively, these parameters covered routine clinical scanning ranges, enabling the evaluation of model stability and reproducibility under heterogeneous imaging conditions.

The study data were collected from glioma patients who received surgical resection at our clinical center (XJYY-GBM) and two public datasets (LUMIERE and RHUH-GBM). All postoperative MRI scans were obtained before tumor recurrence, with imaging time windows specified for each dataset as follows: RHUH-GBM within 72 h post-surgery; LUMIERE between 1 and 3 weeks (≤21 days) postoperatively; XJYY-GBM between 4 and 41 days postoperatively (median: 14 days). Postoperative MRI scans from the three datasets were exclusively used as model inputs, and no scans obtained after tumor recurrence were included.

The postoperative segmentation mask was defined as the visible region on early postoperative T2WI images. The delineation criteria included T2WI hyperintensity, anatomical continuity with the preoperative tumor, and relatively distinct boundaries. Postoperative inflammatory changes and normal tissues surrounding the surgical cavity were excluded during delineation. For the internal dataset (XJYY-GBM), postoperative masks were independently delineated by two neuroradiologists using only early postoperative MRI scans, with no access to follow-up or recurrence images. Recurrence labels were collected separately after mask delineation was completed. For the public datasets (LUMIERE and RHUH-GBM), only masks corresponding to the early postoperative time points were extracted as model inputs.

**Figure 1:**
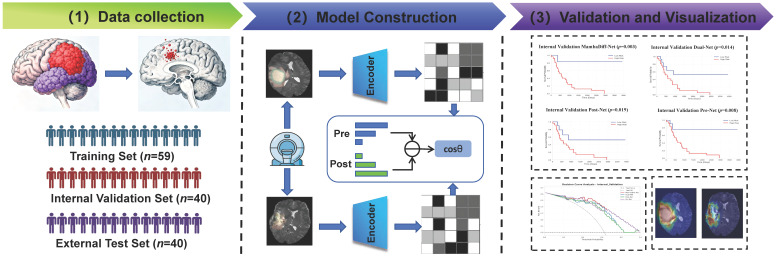
Overall Framework. The workflow of this study includes the following steps: (1) Data acquisition: Collect and preprocess preoperative and postoperative MRI images of patients with glioblastoma from multiple centers, and extract the region of interest (ROI). (2) Model construction: Develop the MambaDiff-Net model based on dual-time-point MRI, integrating a dual-stream encoder and a feature discrepancy computation (FDC) module to model dynamic changes in imaging features. (3) Validation and visualization: Evaluate model performance on internal validation and external test sets, and use Grad-CAM to visualize decision regions associated with recurrence.

In this study, early recurrence was uniformly defined as progression-free survival (PFS) ≤ 6 months. PFS was calculated as the interval from the date of surgery to the first radiologically confirmed recurrence. For the internal dataset (XJYY-GBM), recurrence events and their occurrence times were independently assessed by two neuroradiologists based on follow-up images in accordance with the RANO criteria, and PFS was subsequently computed. Any discrepancies were resolved by consensus. For the LUMIERE dataset, the first time point rated as progressive disease (PD) according to RANO criteria after surgery was taken as the recurrence time for PFS calculation. The PFS labels provided by the RHUH-GBM dataset were adopted directly. Notably, the follow-up images used for label determination were not included in the three datasets. The overall workflow of this study is illustrated in Figure 1.

Inclusion criteria were:

- Pathologically confirmed glioblastoma, WHO grade IV
- Complete preoperative and postoperative data
- Available corresponding tumor segmentation masks

Exclusion criteria were:

- Presence of other primary malignant tumors
- Severe imaging artifacts precluding quantitative analysis
- Incomplete clinical or follow-up data

A total of 139 patients were enrolled, including 40 from the internal clinical center (XJYY-GBM), 59 from the LUMIERE public dataset [30], and 40 from the RHUH-GBM public dataset [31]. The LUMIERE dataset was used as the training set (*n* = 59), the XJYY-GBM dataset as the internal validation set (*n* = 40), and the RHUH-GBM dataset as the external test set (*n* = 40).

### 2.2. Model Overview

**Figure 2:**
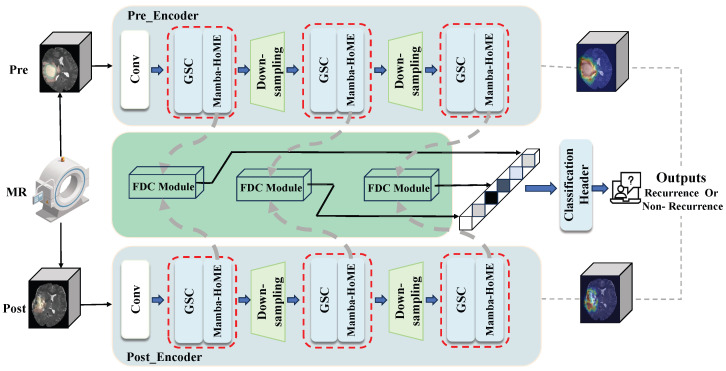
Overall Framework of MambaDiff-Net. After extracting the ROI by multiplying the preoperative and postoperative T2WI images with their corresponding masks, the images are fed respectively into the dual-stream Mamba-HoME encoder for feature extraction. The feature discrepancy computation (FDC) module captures the dynamic changes between preoperative and postoperative images through numerical differences and cosine similarity. Finally, the classification head outputs the recurrence risk probability.

This study proposes MambaDiff-Net (Mamba-based Difference Network), a longitudinal deep learning framework based on preoperative and postoperative dual-time-point MRI for predicting the risk of glioblastoma recurrence. The overall framework is illustrated in Figure 2. By simulating the clinical logic of comparing preoperative and postoperative images, this framework captures longitudinal imaging changes, enabling a transition from static image analysis to dynamic risk assessment.

The model inputs consist of preoperative and postoperative T2WI images along with their corresponding tumor annotation masks. Element-wise multiplication of the images and masks yields region-of-interest (ROI) images, which are then processed by subsequent modules. Specifically, the ROI images are separately fed into two dual-stream Mamba-HoME encoders with identical structures but independent parameters, ensuring feature purity and comparability at each time point. Subsequently, the multi-scale features output by the encoders are processed together in the feature discrepancy computation (FDC) module. By computing numerical differences and cosine similarity between feature maps, this module precisely localizes imaging change regions associated with tumor evolution, thereby capturing the dynamic characteristics induced by surgical intervention. Finally, the fused discrepancy features are passed to the classification head, where they are mapped through fully connected layers to output an individualized recurrence risk probability between 0 and 1.

### 2.3. Dual-Stream Mamba-HoME Encoder

Most existing two-stream networks (e.g., Siamese networks) adopt encoders with weight sharing, which are designed to extract shared features from two inputs. However, in preoperative and postoperative medical image analysis, surgical intervention causes fundamental alterations in tumor regions. Preoperative images reflect the natural growth state of tumors, while postoperative images contain surgical trauma, tissue loss, and inflammatory responses, leading to inherently distinct feature distributions. Forcing the use of weight-shared encoders makes the model prioritize shared patterns over discrepancies, hindering the capture of surgery-induced dynamic changes.

To address this issue, this study proposes a two-stream Mamba-HoME encoder with parameter independence. The two encoders do not share weights, enabling the model to learn respective feature distributions for preoperative and postoperative images, thereby better preserving and amplifying differential information. Specifically, the two-stream Mamba-HoME encoder consists of two parallel branches with identical architectures but independent parameters, each processing preoperative and postoperative ROI images respectively. This parameter-independent design facilitates unbiased learning of time-point-specific features, prevents blurring of temporal differences caused by weight sharing, and ensures subsequent modules focus on surgery-induced biological changes rather than inherent similarities between images. Each encoder branch is built upon the Mamba state space model, which effectively captures long-range spatial dependencies of tumors in MRI scans. Adopting a hierarchical structure, the encoder gradually expands the receptive field via progressive downsampling. Combined with the Global-Local Spatial Convolution (GSC) module, it extracts fine-grained local details (e.g., marginal infiltration) and global morphological traits (e.g., mass effect) of tumors across multiple scales.

To further enhance the representation of the high heterogeneity of glioblastoma, a Hierarchical Soft Mixture of Experts (Hierarchical SoftMoE) layer is integrated at the end of each encoder stage. Glioblastoma is highly heterogeneous, with tumors from different patients showing substantial variations in morphology, boundaries and infiltration patterns. Conventional feedforward networks fail to fully characterize such diversity. SoftMoE assigns each token to multiple experts via a soft gating mechanism, eliminating training instability caused by discrete routing in traditional Mixture of Experts. Its hierarchical design organizes expert groups at different semantic levels: low-level experts focus on fine-grained features such as local textures and edges, while high-level experts handle global characteristics including overall tumor morphology and mass effect. Through dynamic routing and feature refinement across multiple expert networks, the model adaptively captures diverse glioblastoma phenotypes at multiple granularities and boosts representational capacity without a significant increase in computational cost. The multi-scale features output by the two-stream encoders are spatially aligned, laying a solid foundation for layer-wise comparison in subsequent difference modeling.

### 2.4. Feature Discrepancy Computation (FDC) Module

Dynamic differences between pre- and postoperative images directly reflect the extent of surgical resection and characteristics of residual lesions. To effectively capture such discrepancies, a dedicated module for difference modeling is needed, rather than merely stacking or concatenating dual-phase features. Most existing methods calculate numerical differences between feature maps via element-wise subtraction, which can capture only regions with obvious intensity changes, such as newly enhanced lesions. Nevertheless, some recurrence-related alterations manifest as semantic shifts, including variations in texture, morphology and edge features, without remarkable intensity differences. To address this issue, we propose a Feature Discrepancy Computation (FDC) Module to model dynamic imaging changes induced by surgical intervention.

Specifically, this module takes multi-scale features output by the dual-stream encoder as inputs, where preoperative and postoperative features are spatially aligned. For each pair of spatially corresponding feature vectors, the module first computes the element-wise absolute difference to capture intensity variations in tumor regions. Meanwhile, cosine similarity is calculated and converted into a semantic inconsistency weight, where the weight is defined as 1 minus the similarity score, to identify regions with subtle intensity changes but apparent semantic shifts. The element-wise product of numerical differences and semantic weights is then adopted to generate weighted difference feature maps. This design enables the model to simultaneously focus on newly enhanced lesions with prominent numerical differences and potential early infiltration regions with significant semantic shifts.

On this basis, the Top-K pooling strategy is applied to aggregate features from the *K* spatial positions with the most distinct differences, which effectively suppresses background noise. Finally, multi-scale weighted difference features are concatenated and fused into a compact representation vector to characterize tumor dynamic evolution from preoperative to postoperative stages.

### 2.5. Implementation Details

All raw T2WI images underwent systematic preprocessing before being input into the model. First, temporal registration was performed between preoperative and postoperative images to ensure consistent spatial positions across the two time points. The images were then resampled to a uniform volume size of 96×96×64 voxels using trilinear interpolation. Z-score normalization was applied using the global mean and standard deviation calculated from the training set (LUMIERE), and pixel values were clipped to the range [−3,3] to reduce the impact of extreme outliers.

Tumor masks were binarized and resampled to 96×96×64 voxels via nearest-neighbor interpolation. To compensate for discrepancies in segmentation boundaries across datasets, preoperative masks were dilated twice to cover peritumoral regions, and postoperative masks were eroded once to eliminate uncertainties at the edge of the resection cavity. The processed masks were multiplied element-wise with the corresponding images to mask out non-brain tissue.

To enhance data diversity and reduce the risk of overfitting, data augmentation was applied during training, including random flipping, Gaussian noise injection, and brightness and contrast adjustments. Hyperparameters were empirically selected based on the performance of the validation set. The AdamW optimizer was adopted with a learning rate of $3\times 10^{-5}$, weight decay of $5\times 10^{-4}$, $\beta_{1}=0.9$, and $\beta_{2}=0.999$. The learning rate scheduler was CosineAnnealingWarmRestarts with $T_{0}=10$, $T_{\mathrm{mult}}=2$, and $\eta_{\min}=1\times 10^{-6}$. The CombinedLoss (α=0.7), which integrates Focal loss and BCE loss, was used as the loss function. The maximum training epoch was set to 100 with an early stopping patience of 25, the batch size was set to 16, and the gradient clipping threshold was 1.0. The total number of model parameters was approximately 20.60 M. To ensure experimental reproducibility, a fixed random seed of 42 was set and CuDNN’s non-deterministic algorithms were disabled. All experiments were implemented in PyTorch 2.5.0 and conducted on a Tesla V100-PCIE-32GB GPU (NVIDIA, Santa Clara, CA, USA).

### 2.6. Model Evaluation and Statistical Analysis

This study employed multi-dimensional metrics to evaluate the performance of the proposed model in predicting postoperative recurrence of glioblastoma. Grad-CAM was utilized to visualize the model’s decision regions, thereby enhancing model interpretability. The area under the curve (AUC) was calculated from the receiver operating characteristic (ROC) curve. At the optimal threshold determined by the Youden index, the corresponding Accuracy, F1, Precision, and Recall were computed to comprehensively assess the discriminative ability of the model. The calculation formulas for the metrics are as follows:

(1) $$\begin{array}{c} \mathrm{Accuracy}=\frac{\mathrm{TP}+\mathrm{TN}}{\mathrm{TP}+\mathrm{TN}+\mathrm{FP}+\mathrm{FN}} \end{array}$$

(2) $$\begin{array}{c} F1=2\times \frac{\mathrm{Precision}\times \mathrm{Recall}}{\mathrm{Precision}+\mathrm{Recall}} \end{array}$$

(3) $$\begin{array}{c} \mathrm{Precision}=\frac{\mathrm{TP}}{\mathrm{TP}+\mathrm{FP}} \end{array}$$

(4) $$\begin{array}{c} \mathrm{Recall}=\frac{\mathrm{TP}}{\mathrm{TP}+\mathrm{FN}} \end{array}$$

Among these metrics, AUC is the area under the receiver operating characteristic curve; *TP*, *FP*, *TN*, and *FN* represent true positive, false positive, true negative, and false negative, respectively.

Statistical analyses were performed using Python 3.8 (Python Software Foundation, Wilmington, DE, USA). Continuous variables are presented as mean ± standard deviation, while categorical variables are presented as frequency (percentage). Between-group comparisons were conducted using the independent samples *t*-test or chi-square test, with *p* < 0.05 considered statistically significant.

## 3. Results

### 3.1. Clinical Characteristics

To describe the study population comprehensively, we present the baseline clinical characteristics of the patients (Table 1) to illustrate the population composition across the three centers. A total of 139 patients with glioblastoma were enrolled in this study, including 59 in the training set, 40 in the internal validation set, and 40 in the external test set. The patients ranged in age from 42 to 82 years, comprising 79 males (56.83%) and 60 females (43.17%).

All patients were pathologically confirmed to have WHO grade IV glioblastoma and underwent surgical treatment. In clinical research, the six-month postoperative time point is considered an important prognostic landmark. Based on this, patients in the training set (*n* = 59), internal validation set (*n* = 40), and external test set (*n* = 40) were further classified into recurrence and non-recurrence groups according to whether recurrence occurred within six months.

As shown in Table 1, there were significant differences among the three centers in terms of age (*p* < 0.001) and IDH mutation status (*p* = 0.009), while there was no significant difference in gender distribution (*p* = 0.116). These differences reflect the characteristics of patient composition across different medical institutions in multicenter studies.

We conducted subgroup analyses to investigate whether differences in clinical characteristics across datasets would confound the performance assessment of MambaDiff-Net. Given the small sample size of patients with IDH mutation or unknown status (only 0 to 9 mutant cases in each dataset), we re-evaluated model performance in the IDH wild-type subgroup, which constituted 74.1% of the total cohort (103/139 patients). Meanwhile, we performed age-stratified analysis with 60 years of age as the cutoff value. The results are shown in Table 2.

As presented in Table 2, MambaDiff-Net achieved stable performance across all subgroups. For the IDH wild-type subgroup, the internal validation AUC was 0.875 and the external test AUC was 0.778, which were largely consistent with the results of the overall cohort. In the age-stratified analysis, the internal validation AUC was 0.821 for patients aged ≤ 60 years and 0.825 for those aged > 60 years, while the corresponding external test AUC values were 0.741 and 0.756. In the age-stratified analysis, accuracy ranged from 0.740 to 0.769 in both subgroups. Despite significant differences in age distribution and IDH status among the three datasets, no obvious performance decline or bias was observed for MambaDiff-Net across subgroups. These findings demonstrate that disparities in basic clinical characteristics did not substantially confound model evaluation, and the model exhibits favorable generalization and robustness.

### 3.2. Sequence Selection Comparative Experiment

Since the rate of missing data varies across different imaging sequences, multi-modal input leads to a substantial reduction in available samples. Accordingly, we adopted a single-modal input for the model in this study. To identify the optimal input modality, we conducted comparative experiments on four sequences (T1WI, T2WI, T1CE and FLAIR) using only the internal validation set. Cases with poor image quality, missing modalities or incomplete follow-up information were excluded. The final enrollment status and predictive performance of each sequence were presented in Table 3.

As shown in Table 3, the T2WI sequence exhibited the optimal predictive performance (AUC = 0.887 ± 0.075) and the highest data completeness among all compared sequences. Therefore, unimodal T2WI was selected as the model input, and subsequent model training, validation, and testing were performed based on the T2WI sequence.

### 3.3. Model Performance

To validate the effectiveness of the core design of MambaDiff-Net, which is the dynamic difference modeling between preoperative and postoperative images, we conducted multiple sets of comparative experiments.

First, ablation experiments were performed to demonstrate the necessity of the difference modeling strategy. The full model was compared with three variants. (1) Pre-Net: This variant takes only preoperative images as input, with the rest of the architecture identical to MambaDiff-Net. (2) Post-Net: This variant takes only postoperative images as input, with the rest of the architecture unchanged. (3) Dual-Net: This variant uses both preoperative and postoperative images but replaces the difference computation module with feature concatenation, keeping the rest of the architecture unchanged.

Second, to evaluate the incremental value of our method against existing clinical practice and traditional radiomics analysis, we constructed two baseline models. (1) Clinical-Net: This model adopted unified clinical variables available in all three datasets, including age, sex and IDH status, as input features, with logistic regression serving as the classifier. (2) Radiomics-Net: Handcrafted radiomic features were extracted from the ROIs of preoperative and postoperative T2WI images. After Z-score normalization and LASSO feature selection, the selected features were concatenated and fed into the logistic regression classifier.

Third, to further evaluate the superiority of the proposed method, we compared it with current state-of-the-art models, including MVCNN [32], EDCA-Net [33], MedNet [34], TwinCNN [35], and ResNet [36]. The internal validation and external test results are summarized in Table 4, with the best results highlighted in bold.

Additionally, to further assess the statistical significance of the proposed method, we conducted statistical tests on the AUC differences between each comparative model and MambaDiff-Net on the internal validation set and external test set.

In the internal validation set, MambaDiff-Net achieved the optimal overall performance with a high recall of 0.970, indicating a very low false-negative rate and promising clinical value for early recurrence screening. Statistical analysis confirmed that its AUC was significantly higher than those of all comparative models. By comparison, both Pre-Net and Post-Net yielded an AUC of 0.723. Despite favorable precision, they exhibited relatively low recall values of 0.636 and 0.788, respectively, suggesting limited discriminative power of models relying on single-phase imaging. The Dual-Net, which simply concatenated preoperative and postoperative features, achieved a slightly improved AUC of 0.749 but still underperformed MambaDiff-Net, demonstrating that straightforward feature concatenation cannot effectively model dynamic alterations between dual-phase images. In comparison with conventional methods, Clinical-Net (AUC = 0.619) and Radiomics-Net (AUC = 0.679) showed substantially inferior predictive performance, indicating that standalone clinical indicators and traditional handcrafted radiomic features have limited capability for early recurrence prediction. In contrast, the deep learning-based dynamic features learned by MambaDiff-Net offer compelling incremental predictive value. Regarding other deep learning baselines, MVCNN achieved the weakest performance (AUC = 0.674), while EDCA-Net produced relatively better results but remained limited by a low recall of 0.697, implying a considerable risk of missed diagnoses. TwinCNN achieved competitive accuracy (0.850) and F1 (0.909) but a moderate AUC of 0.731, indicating suboptimal ranking ability. The balanced and superior performance of MambaDiff-Net demonstrates that explicitly modeling numerical discrepancies and semantic inconsistencies between preoperative and postoperative images enables comprehensive characterization of continuous tumor progression from preoperative invasion to postoperative residue, thereby substantially improving the accuracy of early recurrence risk prediction.

In the external test set, MambaDiff-Net consistently exhibited the best generalization capability, whereas all comparative models suffered varying degrees of performance degradation. Statistical analyses revealed that the performance superiority of MambaDiff-Net was statistically significant relative to more than half of the comparative models, demonstrating its stable advantages in cross-center scenarios. The AUCs of Pre-Net and Post-Net decreased to 0.685 and 0.701, respectively, further verifying the robustness of our comprehensive model across external datasets. Notably, Dual-Net experienced severe performance degradation, with its recall dropping sharply from 0.697 in the internal validation to 0.515 in the external test, indicating that simple feature concatenation tends to overfit the training distribution and lacks cross-center robustness. Compared with conventional approaches, Clinical-Net and Radiomics-Net yielded reduced AUCs of 0.554 and 0.597, respectively, both inferior to MambaDiff-Net, which further validates the outstanding cross-center generalization of dynamically learned imaging features over traditional methods. Among other baseline models, EDCA-Net maintained relatively stable performance (AUC = 0.751, F1 = 0.764). Unlike the internal validation results, the difference between MambaDiff-Net and EDCA-Net was not statistically significant in the external cohort, which may be attributed to the higher complexity and heterogeneity of cross-center data or the limited sample size of the external test set. In contrast, other baselines including TwinCNN, MVCNN, MedNet, and ResNet all showed degraded AUCs below 0.714; in particular, MVCNN (AUC = 0.584) and MedNet (AUC = 0.541) performed nearly at random guessing levels, further demonstrating their poor robustness against distribution shifts. Remarkably, MambaDiff-Net retained a favorable discriminative AUC of 0.762 under such heterogeneous cross-center conditions, fully highlighting the inherent superiority of the dynamic imaging analysis paradigm for multicenter clinical applications.

Overall, the systematic comparative analysis on the internal validation and external test sets consistently demonstrates that explicitly modeling preoperative-to-postoperative dynamic imaging changes can effectively capture the imaging evolution features associated with early recurrence of glioblastoma, offering significant comprehensive performance advantages and cross-center generalization capability compared to single-time-point analysis or simple feature fusion strategies.

### 3.4. Visual Analysis

**Figure 3:**
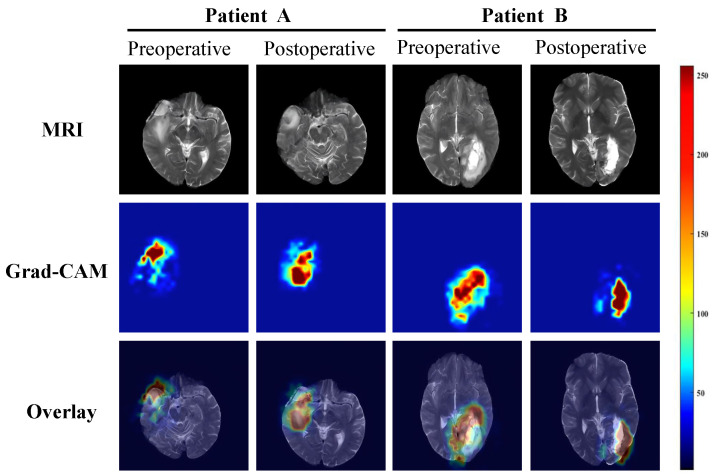
Example of Grad-CAM visualization of the Mamba-Diff Net model. It shows preoperative and postoperative MRI images of a typical case and the corresponding model attention areas.

To further investigate the decision-making basis of the model, this study employed Gradient-weighted class activation mapping (Grad-CAM) to visually analyze the prediction results of MambaDiff-Net. Figure 3 presents the preoperative and postoperative images of representative cases, along with the corresponding attention regions of the model.

As shown in Figure 3, the Grad-CAM activation regions of the model during the preoperative stage are highly concentrated in the solid tumor area, which completely overlaps with the lesion location on MRI, indicating that the model mainly focuses on identifying the core pathological feature of the tumor itself. In the postoperative stage, the activation regions of the model show a significant spatial shift: they no longer strictly correspond to the resected tumor core, but instead shift toward the postoperative resection cavity or potential tumor-infiltrated areas, with a more diffuse distribution of activation intensity. The model exhibits obvious inconsistency in its regions of interest between the preoperative and postoperative stages. This dynamic change intuitively reflects the effect of surgical intervention, shifting from identifying solid tumor lesions to capturing alterations in the postoperative microenvironment. This suggests that the model can sensitively capture dynamic changes in tumor status before and after treatment, providing interpretable visual evidence for risk assessment.

### 3.5. Survival Analysis and Risk Stratification

**Figure 4:**
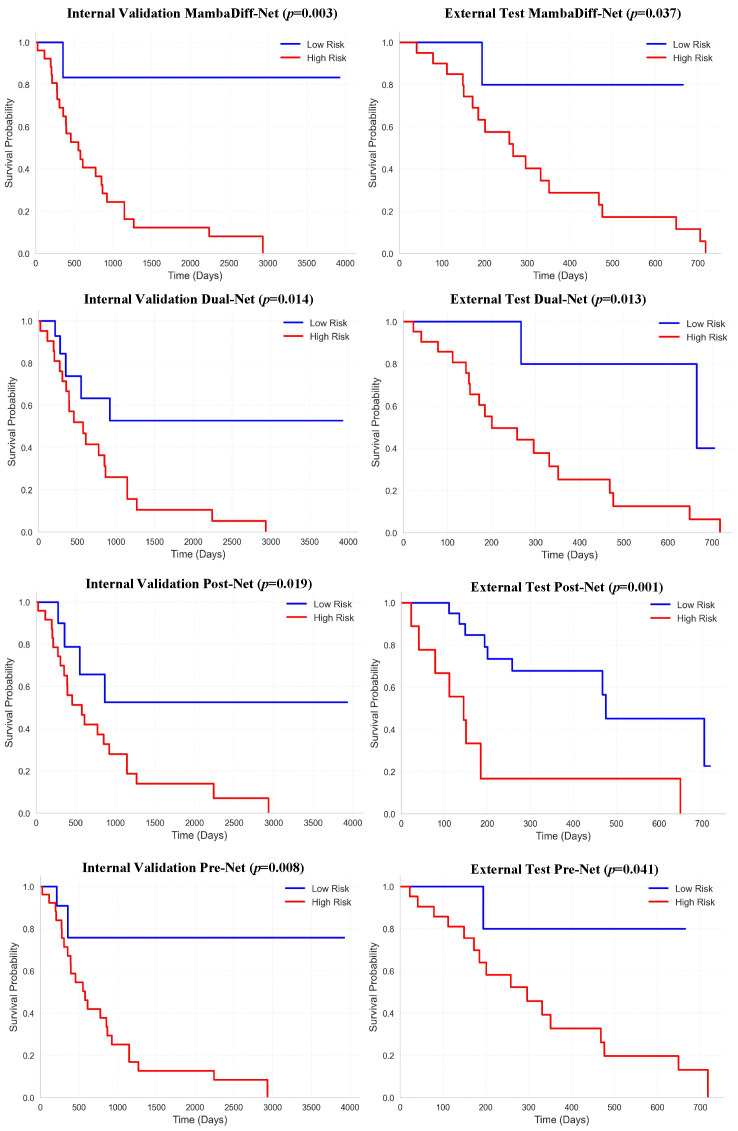
Kaplan-Meier survival curves for the internal validation set (XJYY-GBM) and the external test set (RHUH-GBM). The Kaplan-Meier curves show the risk stratification ability of the three models in the internal validation set (**left**) and the external test set (**right**).

In this study, the Kaplan-Meier survival curves were used to evaluate the risk stratification ability of four predictive models (MambaDiff-Net, Dual-Net, Post-Net, Pre-Net) in the internal validation set and the external test set. Patients were divided into high-risk and low-risk groups based on the predicted risk scores of each model, and the log-rank test was used to compare the survival differences between the two groups. The results are shown in Figure 4.

The Kaplan-Meier survival curves of all models showed that patients in the low-risk group had significantly higher survival probability than those in the high-risk group, and the survival differences between groups were statistically significant. In the internal validation set, the survival curves of the low-risk group for each model remained at a relatively high level, while the survival probability of the high-risk group decreased rapidly over time. This intergroup stratification trend was consistently reproduced in the external test set, indicating that the risk stratification ability of the models had good generalizability. In comparison, MambaDiff-Net demonstrated more robust stratification performance across both validation sets, with the smallest *p*-value in the internal validation set (*p* = 0.003) and a significant difference in the external test set (*p* = 0.037), suggesting that the model has stronger adaptability to different cohorts.

**Figure 5:**
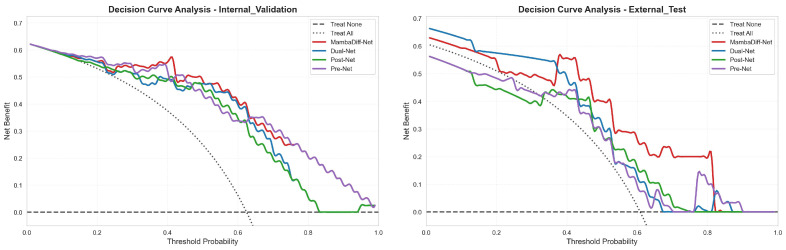
Decision curve analysis (DCA) curves for the internal validation set (XJYY-GBM) and external test set (RHUH-GBM). The DCA curves illustrate the clinical net benefit of the four models in the internal validation set (**left**) and external test set (**right**).

To evaluate the practical clinical utility of the proposed model in clinical decision-making, we performed decision curve analysis (DCA) on the internal validation set and external test set to quantify the clinical net benefit of each model under different threshold probabilities. The results are shown in Figure 5.

In the internal validation set, the net benefit curve of MambaDiff-Net was significantly higher than those of the other models across most threshold probability ranges, particularly within the commonly used low-to-medium threshold range, indicating its ability to more accurately identify high-risk patients without increasing unnecessary interventions. In the external test set, MambaDiff-Net and Dual-Net maintained optimal net benefit performance across a wide range of thresholds, further validating the generalizability of their clinical decision support capabilities. In contrast, Post-Net and Pre-Net exhibited net benefits lower than the “treat all” strategy in certain threshold intervals, suggesting that models based on a single time point have limited clinical decision-making value.

## 4. Discussion

This study proposed and validated MambaDiff-Net, a model that uses dynamic differences between preoperative and postoperative longitudinal MRI to predict the risk of postoperative recurrence in patients with glioblastoma. Unlike most existing studies that are confined to single-time-point static imaging, this model explicitly models the imaging evolution features before and after surgery, achieving a paradigm shift from static description to dynamic assessment. In the internal validation and external test sets, the model achieved AUCs of 0.887 and 0.762, respectively, significantly outperforming single-time-point models and simple feature fusion methods (Table 4). Decision curve analysis (Figure 5) demonstrated that the clinical net benefit of the model consistently exceeded the “treat all” reference line. Kaplan-Meier survival analysis (Figure 4) confirmed its ability to effectively distinguish between high-risk and low-risk patients (internal validation *p* < 0.001, external test *p* = 0.037). Visualization analysis (Figure 3) showed that the model’s focus area shifted from preoperative tumor parenchyma to postoperative resection cavity and peritumoral edema, which is consistent with the clinical patterns of glioblastoma recurrence.

Through the independent-parameter design of the dual-stream encoder, this study implicitly validates a critical yet often overlooked issue in longitudinal imaging analysis: the feature distributions of preoperative and postoperative images may be inherently different and should not be forcibly constrained to the same parameter space. If a weight-sharing encoder were adopted, the model might struggle to learn the dynamic changes induced by surgical intervention, as weight-sharing encoders are naturally inclined to extract common features between the two time points rather than differential features. Preoperative images reflect the natural growth state of the tumor within an intact biological environment, whereas postoperative images are confounded by multiple non-tumor factors, including surgical trauma, tissue loss, inflammatory responses, and repair processes. The signal characteristics of the two time points are substantially different, and the independent parameter encoder provides an algorithmic representation of this clinical reality.

Through the feature discrepancy computation (FDC) module, this study identified two key types of imaging dynamic features: regions with significant numerical differences (corresponding to newly emerged or progressive enhancing lesions) and regions with marked semantic shifts but no obvious changes in signal intensity (corresponding to potential microscopic infiltration areas). This finding suggests that imaging signals of postoperative recurrence risk are not limited to visibly apparent enhancing lesions but also exist in regions where semantic features have shifted without obvious changes in appearance. In contrast, single-time-point models lack the ability to capture changes in such regions, which explains why the recall of static models in this study was significantly lower than that of the complete model (Table 4).

The AUC of our model on the external test set (0.762) was lower than that on the internal validation set (0.887), mainly due to the substantial distribution shift between the training set and the external test set. As presented in Table 1, significant differences in age and IDH status were observed between the two datasets. Additionally, the time points for postoperative MRI acquisition were inconsistent (within 72 h vs. 1–3 weeks). These discrepancies posed a rigorous test for the model’s generalization performance. Nevertheless, the AUC of 0.762 was still considerably higher than that of most comparative models, demonstrating the model’s reliable predictive performance. In future work, domain adaptation techniques or expansion of diverse training datasets could be adopted to further reduce the performance gap across different centers.

Compared with previous prediction studies based on single-time-point imaging, the proposed method has significant advantages. First, existing studies are mostly confined to preoperative or postoperative static imaging analysis [37,38]. Although radiomic features have been demonstrated to be associated with patient prognosis, such methods cannot reflect the impact of surgery, a key interventional event, on the trajectory of tumor evolution. Our findings are consistent with recent literature emphasizing the value of longitudinal imaging analysis [21,22], which suggests that integrating multi-time-point data to describe tumor phenotypic evolution is significantly superior to single-time-point analysis. Second, this study distinguishes between two approaches for utilizing multi-time-point information: feature concatenation and feature discrepancy. Ablation experiments demonstrate that discrepancy modeling is significantly superior to simple concatenation in cross-center generalization performance (Table 4), a finding that provides a methodological reference for future longitudinal imaging studies [39]. Third, this study reveals, through Grad-CAM visualization, the dynamic shift of the model’s focus area from preoperative tumor parenchyma to the postoperative resection cavity margin, providing intuitive evidence for the interpretability of deep learning models in medical imaging analysis (Figure 3).

This study adopted a pure imaging model without incorporating clinical variables such as age and IDH status, primarily to enhance cross-center generalization. Given the substantial heterogeneity in age and IDH status across the three datasets (Table 1, direct inclusion of these variables may introduce center-related bias and impair the model’s cross-center generalization performance. Subgroup analyses revealed (Table 2) that MambaDiff-Net achieved stable performance across IDH wild-type cases and different age subgroups, indicating that variations in basic clinical characteristics did not substantially confound model evaluation and verifying the strong generalization robustness of the pure imaging model. In addition, the Clinical-Net established in this study—which only incorporated age, sex and IDH status—yielded AUC values ranging from 0.58 to 0.62 across all datasets, considerably lower than those of the proposed model (0.887 for internal validation and 0.762 for external validation). This result does not negate the overall prognostic value of clinical information. Instead, this is mainly because Clinical-Net failed to include well-recognized strong prognostic factors, such as resection extent, KPS score and MGMT status. This also reflects the practical challenges in standardizing clinical data for multicenter retrospective studies. As a routine imaging sequence, T2WI does not rely on standardized clinical data that are often difficult to acquire. Therefore, even when key clinical information is unavailable, reliable early recurrence prediction can be realized solely using routinely accessible dynamic preoperative and postoperative imaging features, endowing the model with promising clinical applicability.

Nevertheless, this study still has several limitations. First, the sample size is relatively limited (139 cases), which inherently carries risks of overfitting and reduced model stability. To alleviate these issues, we adopted multiple strategies, including a lightweight network architecture, early stopping, gradient clipping, weight decay, data augmentation and rigorous dataset partitioning. Even with these measures, the small sample size may still cause the model to overlearn noise or accidental patterns in the training set, thus impairing its generalization reliability in real clinical scenarios. In particular, the predictive performance for rare subgroups such as IDH-mutant cases could be constrained. Accordingly, the limited sample size remains a major limitation of this study. Future work will involve enrolling more multicenter samples for further validation. Second, this study only used a single T2WI sequence and did not integrate other clinically valuable imaging modalities such as T1CE and FLAIR. Although internal comparative experiments demonstrated that T2WI achieved better predictive performance than T1CE, multi-sequence fusion can still provide complementary information. In future work, we will explore multi-sequence longitudinal analysis to further improve prediction accuracy. Third, the net benefit of the model in decision curve analysis showed limited advantages compared with the “treat all” strategy in certain threshold intervals, which may be related to the extremely high postoperative recurrence risk of glioblastoma (>90%). Future efforts could explore multi-class risk stratification to more precisely guide clinical decisions. Fourth, this model requires complete preoperative and postoperative T2WI images and corresponding tumor annotation masks. In real-world clinical scenarios, some patients may not be suitable for this model due to poor quality of early postoperative images (e.g., motion artifacts, metal artifacts) or lack of professional annotations. Future research could explore weakly supervised or self-supervised learning strategies to reduce dependence on high-quality pixel-level annotations. Fifth, due to the inherent limitations of multicenter retrospective data, certain clinical and molecular information (e.g., tumor volume, treatment regimens, and MGMT status) was incomplete or inconsistently recorded across the datasets, and thus was not incorporated into our model analysis. Although our study demonstrated that the imaging-only model achieves independent prognostic predictive value, whether the integration of additional clinical and molecular features can further improve model performance remains to be explored in future multicenter cohorts with standardized and comprehensive clinical annotations.

## 5. Conclusions

In this study, we propose MambaDiff-Net, a deep learning model based on preoperative and postoperative longitudinal MRI that quantifies dynamic imaging changes before and after surgery through a dual-stream encoder and a feature discrepancy computation module, enabling accurate prediction of postoperative recurrence risk in patients with glioblastoma. The model achieved AUCs of 0.887 and 0.762 on the internal validation set and external test set, respectively, significantly outperforming single-time-point models and simple feature fusion methods. Kaplan-Meier survival analysis demonstrated that the model effectively distinguishes high-risk from low-risk patients, and decision curve analysis confirmed its robust clinical net benefit. Grad-CAM visualization revealed that the model’s focus area shifts from the preoperative tumor parenchyma to the postoperative resection cavity margin, which is consistent with the clinical and pathological patterns of glioblastoma recurrence. This model provides a feasible pathway for transitioning from static image analysis to dynamic risk assessment and holds promise for assisting clinicians in developing personalized postoperative adjuvant treatment strategies and follow-up plans.

## Figures and Tables

**Table 1:** Comparison of clinical characteristics of the training set, internal validation set, and external test set.

Characteristic	Training		Internal Validation		External Test		*p*
	**(*n* = 59)**		**(*n* = 40)**		**(*n* = 40)**		
	**Recurrence**	**Non-** **Recurrence**	**Recurrence**	**Non-** **Recurrence**	**Recurrence**	**Non-** **Recurrence**	
	**(*n* = 40)**	**(*n* = 19)**	**(*n* = 33)**	**(*n* = 7)**	**(*n* = 22)**	**(*n* = 18)**	
Age (years)	61.6±9.5	60.4±8.9	50.2±8.0	52.4±14.4	62.8±8.5	63.2±10.2	<0.001
Gender							0.116
Male	20 (50.0%)	9 (47.4%)	17 (51.5%)	5 (71.4%)	15 (68.2%)	13 (72.2%)	
Female	20 (50.0%)	10 (52.6%)	16 (48.5%)	2 (28.6%)	7 (31.8%)	5 (27.8%)	
IDH state							0.009
Mutant	1 (2.5%)	0 (0.0%)	9 (27.3%)	0 (0.0%)	3 (13.6%)	1 (5.6%)	
Wild	26 (65.0%)	14 (73.7%)	21 (63.6%)	6 (85.7%)	19 (86.4%)	17 (94.4%)	
Unknown	13 (32.5%)	5 (26.3%)	3 (9.1%)	1 (14.3%)	0 (0.0%)	0 (0.0%)	

**Table 2:** Subgroup analysis for internal validation set and external test set.

Model	N	Internal Validation					External Test				
		**AUC**	**Acc**	**F1**	**Precision**	**Recall**	**AUC**	**Acc**	**F1**	**Precision**	**Recall**
All cases	139	0.887	0.925	0.955	0.941	0.970	0.762	0.800	0.871	0.931	0.818
IDH Wild-type	103	0.875	0.882	0.931	0.964	0.900	0.778	0.833	0.800	1.000	0.667
Age ≤ 60	78	0.821	0.740	0.800	1.000	0.667	0.741	0.769	0.727	1.000	0.571
Age > 60	61	0.825	0.769	0.800	0.750	0.857	0.756	0.769	0.786	0.764	0.813

**Table 3:** Sequence comparison results.

Sequence	Cases (Train/Val/Test)	AUC	Acc	F1	Precision	Recall
T1WI	57/38/40	0.854 ± 0.115	0.816 ± 0.091	0.881 ± 0.089	0.963 ± 0.018	0.813 ± 0.096
T2WI	59/40/40	0.887 ± 0.075	0.925 ± 0.059	0.955 ± 0.047	0.941 ± 0.053	0.970 ± 0.014
T1CE	57/21/40	0.852 ± 0.096	0.857 ± 0.093	0.909 ± 0.043	1.000 ± 0.002	0.833 ± 0.090
FLAIR	57/30/40	0.840 ± 0.101	0.867 ± 0.077	0.913 ± 0.063	0.955 ± 0.027	0.875 ± 0.071

*Note:* All metrics (AUC, accuracy, F1, precision, recall) are bounded between 0 and 1. For values reported as 1.000, the upper bound of the confidence interval is truncated at 1.000.

**Table 4:** Comparison of performance of different models on internal validation set and external test set.

Model	Internal Validation					External Test				
	**AUC**	**Acc**	**F1**	**Precision**	**Recall**	**AUC**	**Acc**	**F1**	**Precision**	**Recall**
Pre-Net	0.723 *	0.675	0.764	0.955	0.636	0.685 *	0.700	0.727	0.750	0.706
Post-Net	0.723 *	0.775	0.852	0.929	0.788	0.701 *	0.733	0.714	0.909	0.588
Dual-Net	0.749 *	0.725	0.807	0.958	0.697	0.714	0.600	0.680	1.000	0.515
Clinical-Net	0.619 *	0.375	0.390	1.000	0.242	0.554 *	0.467	0.273	0.600	0.177
Radiomics-Net	0.679 *	0.775	0.873	0.816	0.939	0.597 *	0.600	0.714	0.600	0.882
MVCNN [32]	0.674 *	0.667	0.667	0.769	0.588	0.584 *	0.600	0.667	0.631	0.706
EDCA-Net [33]	0.801 *	0.750	0.821	1.000	0.697	0.751	0.675	0.764	0.955	0.636
MedNet [34]	0.615 *	0.775	0.857	0.900	0.818	0.541 *	0.575	0.679	0.900	0.545
TwinCNN [35]	0.731 *	0.850	0.909	0.909	0.909	0.688 *	0.675	0.772	0.917	0.667
ResNet [36]	0.761 *	0.675	0.755	1.000	0.606	0.714	0.675	0.764	0.955	0.636
MambaDiff-Net	0.887	0.925	0.955	0.941	0.970	0.762	0.800	0.871	0.931	0.818

*Note*: * indicates that the AUC difference between the comparative model and MambaDiff-Net is statistically significant (p<0.05).

## Data Availability

The original data presented in the study are openly available as follows: the LUMIERE dataset is openly available in FigShare at https://doi.org/10.6084/m9.figshare.c.5904905; the RHUH-GBM dataset is openly available in TCIA at https://doi.org/10.7937/4545-c905. The data presented in this study for the XJYY-GBM dataset are available on request from the corresponding author due to restrictions imposed by the institutional ethics committee and the data protection policy of the affiliated hospital.
